# Arhovin Internally in Gonorrhea of the Male

**Published:** 1909-12

**Authors:** 


### Arhovin Internally in Gonorrhea of the Male.

Dreysel, Leipzig, (*Fortschritte der Medizin*, February 10, 1908), used arhovin in fifty-eight ambulant gonorrhea cases, forty-seven acute, eight subacute, three chronic. It is doubtless borne better than any other medicament. He gave it for periods of six weeks and longer, in some cases ten to twelve capsules daily, but never saw any alimentary disturbance; he never had to suspend its exhibition, though some of his patients had previously rebelled against gonosan and santyl. The significant oral odor which often follows use of the balsams never appears with arhovin. Never was there pressure in the renal region or albuminuria. Even sensitive and irritable bladders tolerated it perfectly.

Subjective difficulties in the anterior urethra, burning on micturition, painful erections, are generally influenced most favorably by arhovin, though when there are severe inflammatory symptoms its analgesic action is sometimes less striking. Exceptionally the pains persisted even for two or three weeks. In affections of the posterior urethra, the effect of arhovin on subjective symptoms was even more evident. Burning, tenesmus, pains in the region of the bladder, generally soon ceased, often in a few days, only exceptionally persisting for two or three weeks. Urinary acidity was enhanced by arhovin; when the urine was but feebly acid and cloudy, arhovin often cleared it up.

To determine the prophylactic action of arhovin, Dreysel used it in twenty-three acute gonorrheas in which the inflammation was strictly limited to the anterior urethra. In only six of them did cloudiness of the urine appear; in two it was very slight, subjective difficulties being almost entirely absent. Of the four others an epididymitis and prostatitis appeared in one. Metastatic formation, especially joint affections, did not appear once. The usual percentage of posterior involvement is 80 to 90 per cent. and the fact that in these cases it was but 26 per cent. must be ascribed to arhovin. The urine rendered bactericidal by arhovin prevents spreading of the gonorrheal process. One patient who, under arhovin, had remained free from complications for three weeks, took it upon himself to discontinue it; shortly afterwards a posterior urethritis and epididymitis set in. This prophylactic efficacy of arhovin, Dreysel thinks, is its most valuable property, since the avoidance of complications means a material shortening of the course of the disease and a great simplification of therapy.

Dreysel concludes: Arhovin, like all of the other internal remedies, is no specific against the gonococci; nor is it a panacea for the different symptoms of the process; generally a gonorrhea cannot be cured with arhovin alone. But it is a very valuable adjuvant that is perfectly tolerated, which,—used simultaneously with local measures,—alleviates the difficulties and tends to prevent complications; and which ofttimes cures posterior affections, and that the more certain the less intense is the inflammation and the less extensive is the prostatic involvement.

